# Skull Bone Regeneration Using Chitosan–Siloxane Porous Hybrids—Long-Term Implantation

**DOI:** 10.3390/pharmaceutics10020070

**Published:** 2018-06-08

**Authors:** Yuki Shirosaki, Motomasa Furuse, Takuji Asano, Yoshihiko Kinoshita, Toshihiko Kuroiwa

**Affiliations:** 1 Faculty of Engineering, Kyushu Institute of Technology, 1-1 Sensui-cho, Tobata-ku, Kitakyushu 804-8550, Japan; 2 Department of Neurosurgery, Osaka Medical College, 2-7 Daigaku-machi, Takatsuki, Osaka 569-8686, Japan; neu054@poh.osaka-med.ac.jp (M.F.); neu040@poh.osaka-med.ac.jp (T.K.); 3 Nikkiso Co., Ltd., Ebisu, Shibuya-ku, Tokyo 150-6022, Japan; takuji.asano@nikkiso.co.jp (T.A.); y.kinoshita@nikkiso.co.jp (Y.K.)

**Keywords:** skull bone regeneration, chitosan-siloxane porous hybrid, long-term implantation

## Abstract

Burr holes in craniotomy are not self-repairing bone defects. To regenerate new bone at the sites of these defects, a good scaffold is required. Biodegradable hybrids including silica or siloxane networks have been investigated as bone tissue scaffolds. This study examined skull bone regeneration using chitosan-siloxane hybrids after long-term implantation (two and three years). After implantation of the hybrids, the surrounding cells migrated and formed fibrous tissues and blood vessels. Then, bone formation occurred from the surrounding blood vessels. Addition of calcium ions and coating with hydroxyapatite improved bone regeneration. Finally, the regenerated tissue area became smaller than the initial hole, and some areas changed to completed bone tissues.

## 1. Introduction

“Burr holes” are required in craniotomy to insert surgical tools for neurological and brain surgeries [1,2,3]. They often leave undesirable indentations in the area, and patients are unsatisfied after surgery, even in areas where there is abundant coverage by hair. To cover the holes, various materials have been used, such as metallic plates [4,5], hydroxyapatite buttons [6,7,8,9], calcium phosphate cements [10,11,12], and acrylic cements [13,14]. Chronic pain is caused by metallic plates, and thinning of the skin also occurs, leading to extrusion of the plate [15,16]. Moreover, the metallic plates interfere with magnetic resonance imaging. In the case of acrylic cement, irritation is caused by the exothermic heat of polymerization [13,14]. Hydroxyapatite buttons are good candidates for burr holes because of their osteocompatibility. However, they are not flexible, and it is difficult to adjust their shapes to the defects during the operation. Calcium phosphate cements are also compatible with bone formation, but they also have the risks of leakage into the brain before hardening because of their delayed setting time [17]. Thus far, many biomaterials have been used for facial bone regeneration [18].

Biodegradable organic-inorganic hybrids have been investigated as bone tissue scaffolds because of their controllable degradation and flexibility. Many studies have reported that hybrids including silica or silicate improve osteocompatibility and bone formation [19]. Hybrids with siloxane networks have also been investigated for their ability to promote bone formation. However, in vivo examination has been insufficient to clarify the effect of their structure on bone regeneration. 

In previous studies [20,21,22,23,24], we investigated a chitosan-γ-glycidoxypropyltrimethoxysilane (GPTMS) hybrid prepared by the sol-gel method. The human osteosarcoma cell line MG63 adhered and proliferated on the hybrid membranes and showed high alkaline phosphatase activity compared with the chitosan membrane [20,21]. Moreover, human osteoblast bone marrow cells differentiated and formed a fibrillar extracellular matrix with numerous calcium phosphate globules [21]. MG63 cells also migrated and proliferated into the pores of porous hybrids prepared from the same sols [22]. These results indicate that hybrids have the potential for use as bone tissue scaffolds. We also prepared chitosan-GPTMS porous hybrids with hydroxyapatite (HAp) by soaking in an alkaline phosphate solution and observed skull bone formation in vivo [24]. The blood from burr holes infiltrated the pores and prevented overflowing. No inflammation occurred, calcium ions were incorporated into the hybrids, and the hydroxyapatite modified on their surfaces accelerated new bone formation during one year, however, the bone formation was not yet completed.

In this study, we observed bone formation in long-term implantations, i.e., for two and three years, to clarify the potential of chitosan–siloxane hybrids for skull bone regeneration.

## 2. Materials and Methods 

### 2.1. Preparation of Porous Hybrids

The porous hybrids were prepared by previously published methods [24]. Chitosan (0.5 g, high molecular weight, deacetylation: 79.0%, Aldrich^®^, St. Louis, MO, USA) was dissolved in aqueous acetic acid (0.25 M, 25 mL). GPTMS (Lancaster, Lancashire, UK) and calcium chloride (Nacalai Tesque, Kyoto, Japan) were added to the chitosan solution to provide a molar ratios of chitosan-GPTMS (ChG) of 1.0:0.5 and chitosan-GPTMS-CaCl_2_ (ChGCa) of 1.0:0.5:1.0. One mole of chitosan equates to one mole of deacetylated amino groups. The mixtures were stirred for 1 h at room temperature, and fractions of each resultant sol were poured into a polystyrene container and frozen at −20 °C for 24 h. The frozen sols were then transferred to a freeze dryer (FDU-506, EYELA, Tokyo, Japan) for 12 h until dry. The obtained porous ChG and ChGCa hybrids were then washed with NaOH (0.25 M) and distilled water to neutralize the remaining acetic acid and lyophilized again. Some ChGCa hybrids were not washed with NaOH but were soaked in aqueous Na_2_HPO_4_ (0.01 M, pH 8.8) at 80 °C for 3 days (ChGCa_HAp). The hybrids were then washed with distilled water and lyophilized again. The compressive strength was measured by a creep meter (RE2-3305C, YAMADEN Co., Ltd., Tokyo, Japan). The compressive test was carried out at 0.5 mm/s to calculate the compressive stress at a 50% strain rate.

### 2.2. In Vivo Animal Experiments

The hybrids were sterilized by the ethylene oxide gas method and maintained for one week at room temperature to clear any ethylene oxide gas remnants. All surgical procedures were performed with the approval of the animal care and use committee of Osaka Medical College (Approval No. 26044). In brief, eight adult female beagles weighing around 10 kg were anesthetized with a mask, using isoflurane (Abbott, Tokyo, Japan) in oxygen. Tracheal intubation was performed after anesthesia induction. Anesthesia was maintained by isoflurane in oxygen delivered via a calibrated vaporizer (TEC3, Ohmeda, UK). The beagles were cleaned and draped in a standard manner with a longitudinal incision made over the scalp, and the pericranium was lifted off with a sharp periosteal elevator. Then, four 10 mm burr holes were created in each beagle dog using a drill to evacuate the liquefied hematoma. After evacuation of the hematoma, a small wound was created on the dura mater, and the hybrids were inserted into each burr hole. Commercial bone cement was also implanted as a control. The periosteal and skin were sutured. All of the above surgical procedures were performed in a sterile manner. Specimens of the parietal bones, including the cranioplasty, were harvested and examined histologically. After fixation in 10% buffered formaldehyde, the specimens were dehydrated in a series of ethanol solutions and soaked in xylene for defatting. Subsequently, the specimens were decalcified, and thin sections were prepared by a microtome and stained with hematoxyline-eosine (HE) and Azan-Mallory (AM) method. The AM staining method is commonly used to distinguish cells from extracellular components and stains muscle fibers red, while cartilage and bone matrix are stained blue [25]. 

## 3. Results and Discussion

**Figure 1:**
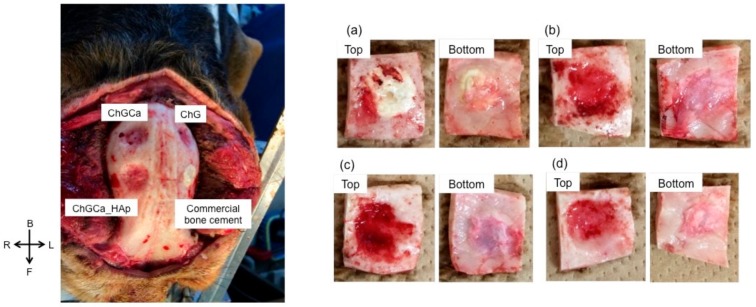
Photographs of Beagle skulls implanted with the samples after three years. On the left, the general appearance of a skull is shown, and, on the right, the appearance of each implanted position is shown. (**a**) commercial bone cement; (**b**) chitosan–γ-glycidoxypropyltrimethoxysilane (GPTMS) (ChG); (**c**) chitosan–GPTMS–CaCl_2_ (ChGCa); and (**d**) chitosan–GPTMS with hydroxyapatite (HAp) (ChGCa_HAp).

The defect size (10 mm) is critical in the beagle dog skull and remains even after seven years, indicating slow self-healing. Figure 1 shows the appearance of the skull implanted with samples after three years. All beagles survived and no infection or auto-mutilation were observed. No inflammation was also observed visually. The dura mater recovered after implantation in all samples. The commercial bone cement performed the same as the hybrids after one year from the implantation [24], and the interface between the bone cement and bone tissue was very clear. Chitosan hybrids (ChG, ChGCa, and ChGCa_HAp) did not remain the same, and new tissues regenerated. In the case of the hybrids, the holes became smaller compared to their initial size, and a hollow in the implanted site was observed. The implanted site of ChGCa_HAp was harder than that of ChG and ChGCa upon palpation.

**Figure 2:**
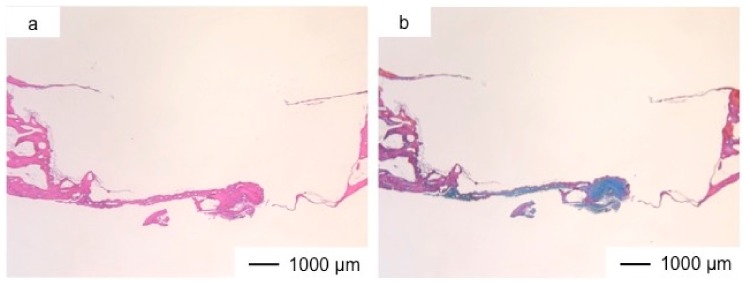
Light microscopic images of the commercial bone cement in cranioplasty at three years postoperatively stained by hematoxyline–eosine (HE) (**a**) and Light microscopic images of the commercial bone cement in cranioplasty at three years postoperatively stained by Azan-Mallory (AM) method (**b**).

**Figure 3:**
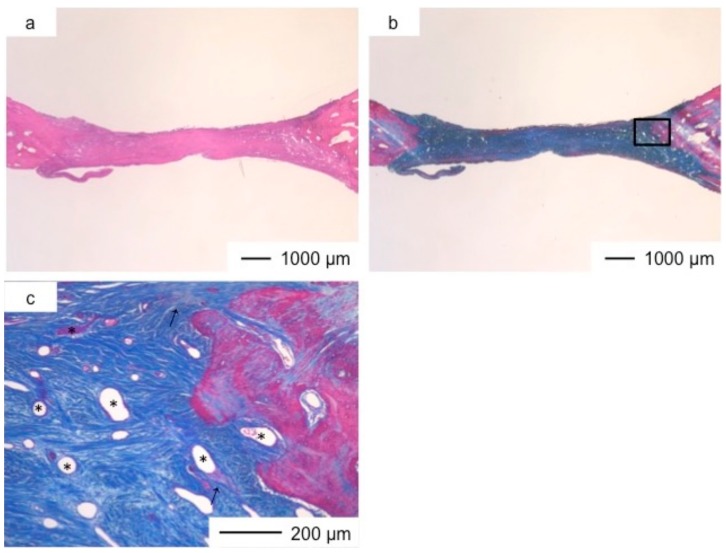
Light microscopic images of ChG in cranioplasty two years after implantation, stained by HE (**a**); and AM (**b**); (**c**) Magnification of the inset in (**b**).

**Figure 4:**
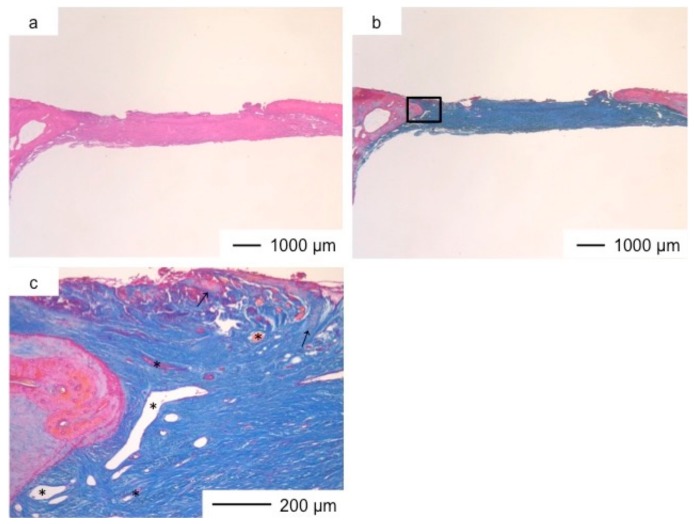
Light microscopic images of ChG in cranioplasty three years after implantation, stained by HE (**a**) and Light microscopic images of ChG in cranioplasty three years after implantation, stained by AM (**b**); (**c**) Magnification of the inset in (**b**).

**Figure 5:**
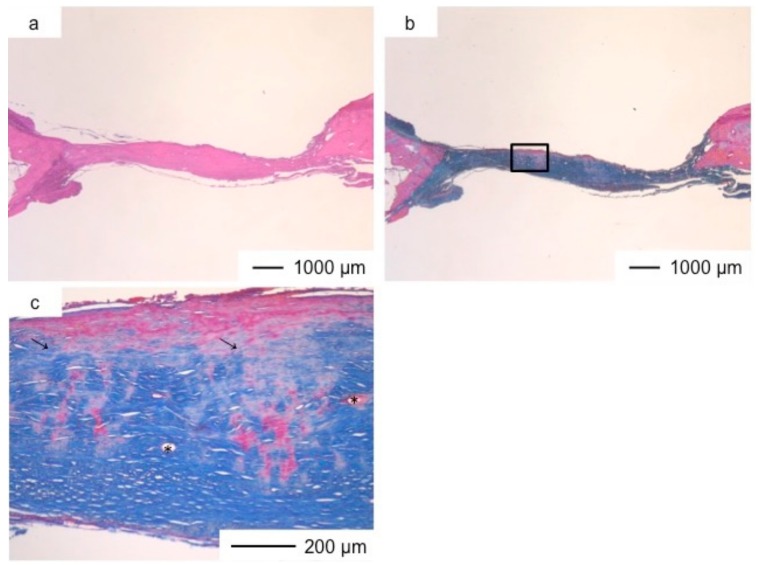
Light microscopic images of ChGCa in cranioplasty two years after implantation, stained by HE (**a**) and Light microscopic images of ChGCa in cranioplasty two years after implantation, stained by AM (**b**); (**c**) Magnification of the inset in (**b**).

**Figure 6:**
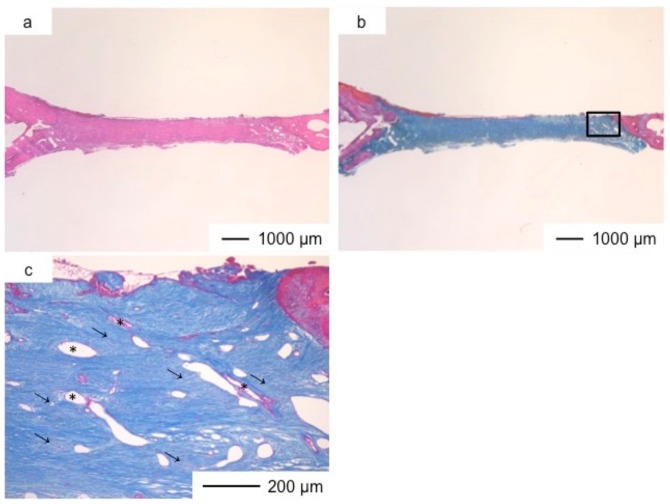
Light microscopic images of ChGCa in cranioplasty three years after implantation, stained by HE (**a**) and Light microscopic images of ChGCa in cranioplasty three years after implantation, stained by AM (**b**); (**c**) Magnification of the inset in (**b**).

**Figure 7:**
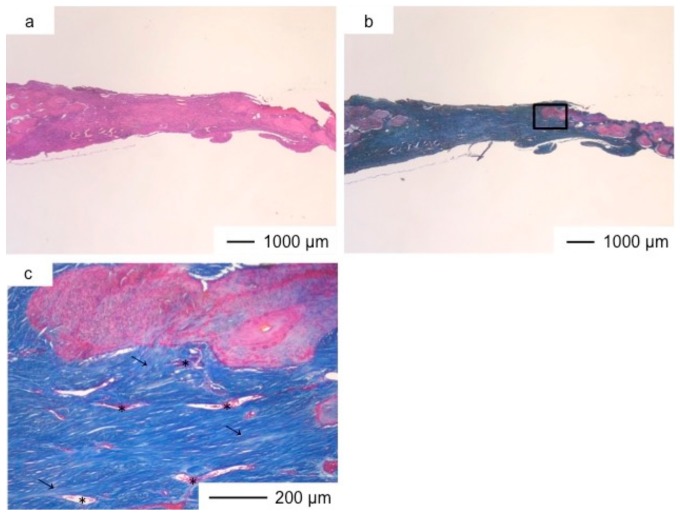
Light microscopic images of ChGCa_HAp in cranioplasty two years after implantation, stained by HE (**a**) and Light microscopic images of ChGCa_HAp in cranioplasty two years after implantation, stained by AM (**b**); (**c**) Magnification of the inset in (**b**).

**Figure 8:**
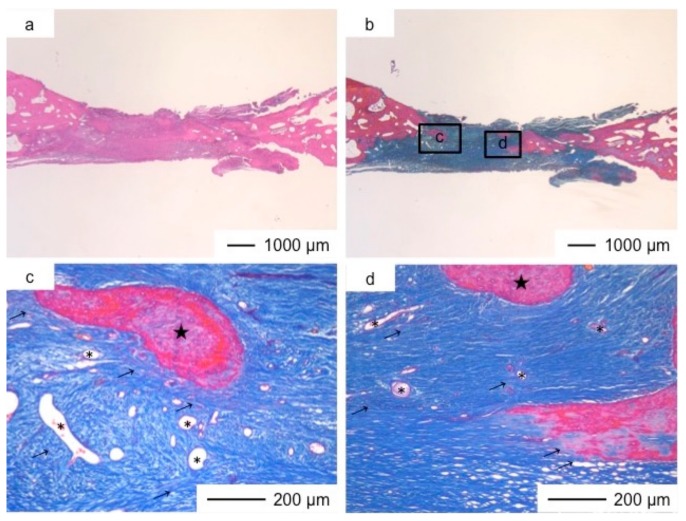
Light microscopic images of ChGCa_HAp in cranioplasty three years after implantation, stained by HE (**a**) and Light microscopic images of ChGCa_HAp in cranioplasty three years after implantation, stained by AM (**b**); (**c**,**d**) Magnifications of the insets in (**b**).

Figure 2, Figure 3, Figure 4, Figure 5, Figure 6, Figure 7 and Figure 8 show the histological results postoperatively. The commercial bone cement did not change the bone defect size even after three years, as shown in Figure 2. At the bottom of the cement, fibrous tissues had regenerated to reconstruct the dura mater. The bone cement inhibited the regeneration of new tissues, and the top of the implanted site was still open. 

Porous hybrid materials had degraded completely and were substituted by fibrous and bone tissues after one year [24]. No defect was observed at the implanted site in histological images (Figure 3, Figure 4, Figure 5, Figure 6, Figure 7 and Figure 8), such as those observed in sites implanted with the commercial bone cement. The regenerated tissues in ChG and ChGCa implantations were about 1 mm in thickness, and the thickness did not increase after theee years (Figure 3, Figure 4, Figure 5 and Figure 6). There were many blood vessels (*) with havers canals in the newly formed tissue, and osteoid tissue formation and new bone matrix (→) were observed around the blood vessels. The defect size of ChG (Figure 3 and Figure 4) and ChGCa (Figure 5 and Figure 6) implantations did not change between two and three years after implantation. The formation of new bone matrix in the osteoid tissues of the ChGCa implantation was much more accelerated than that in the site of the ChG implantation. New bone formation occurred even in the middle and upper area of the implanted site (Figure 5c).

In previous studies [20,21], we confirmed that human osteosarcoma cells and bone marrow cells proliferated on or into a chitosan–siloxane hybrid (ChG) and showed good alkaline phosphate activity. Other studies have reported that porous chitosan has healing properties [26,27] because it takes up wound exudates and forms blood vessels to promote tissue regeneration. In general, connective tissue regeneration follows fibroblast proliferation and extracellular matrix synthesis [28]. Connective tissues regenerated at the implanted sites of chitosan-siloxane hybrids. Tissue regeneration induced by the hybrids occurred as follows; (1) blood filtrated into the pores of the hybrids; (2) fibroblast proliferation was stimulated in the hybrids; (3) fibroblasts synthesized extracellular matrix; (4) blood vessels formed in the regenerated tissues; (5) osteoblasts in the tissues secreted osteoid; (6) osteoid became mineralized to form new bone tissues. Soluble silica and calcium ions stimulate bone growth [29]. The silicon species released from the hybrids also affect osteoblast differentiation [30] and lead to osteoid production in ChG. The higher new bone formation of ChGCa depends on calcium ions released from the hybrids. However, the connective tissues size was unchanged, indicating that calcium ions released from the hybrids promoted the formation of new bone matrix by the osteoblasts in the connective tissue. 

The thickness of the tissues regenerated by ChGCa_HAp increased, and the defected size became smaller. The thicknesses after two and three years were about 1.5 and 2.0 mm, respectively (Figure 7 and Figure 8). High new bone formation in osteoid tissues was found in ChGCa_HAp implantations. Not only the pre-existing bone tissues migrated to the implanted site, but also new bone formation (★) occurred in the newly formed tissues (Figure 8c,d). ChGCa_HAp has low crystalline needle-like apatite deposits on the surface of pores [31]. The low crystalline apatite dissolves in vivo, and the released calcium and phosphate ions accelerate bone formation. As a result, bone formation in the ChGCa_HAp implantation was observed in the connective tissue. The decrease in the defect size indicated that the surrounding bone also migrated into the hybrids. 

Although the defects were filled by the newly generated tissue with blood vessels, osteoid, and new bone formation, and the size became smaller, even ChGCa_HAp could not achieve completed bone formation after three years. Engler et al. showed that the elasticity of materials affects mesenchymal stem cell differentiation [32]. Soft matrices are neurogenic, stiffer matrices are myogenic, and rigid matrices induce osteogenesis. In this study, ChGCa_HAp (compressive stress: 0.099 ± 0.007 MPa) was more rigid than the other hybrids (ChG: 0.024 ± 0.008 MPa; ChGCa: 0.034 ± 0.007 MPa). However, it was not stiffer than the natural skull bone. To form completed new bone earlier, the hybrid stiffness should be controlled by crosslinking between chitosan, GPTMS, and hydroxyapatite deposits. 

## 4. Conclusions

Long-term skull bone regeneration was observed using chitosan–siloxane porous hybrids. After two and three years from implantation, commercial bone cement was still present. The hybrids were substituted with new regenerated tissues that were completely closed the holes. Incorporated calcium ions and the inclusion of hydroxyapatite accelerated the new bone formation. However, the thickness of the regenerated skull bone was lower than the normal thickness, and depressions were observed. The results in this study may facilitate the design of new bone tissue scaffolds using hybrids including siloxane units.
